# A phase I clinical study to assess safety and immunogenicity of yellow fever vaccine

**DOI:** 10.1038/s41541-022-00595-6

**Published:** 2022-12-19

**Authors:** Sajjad Desai, K. Anil, Anirudha Vyankatesh Potey, Y. Sindhu, Silvia Grappi, Giulia Lapini, Satyaprasad Manney, Parikshit Tyagi, Emanuele Montomoli, Cyrus S. Poonawalla, Prasad S. Kulkarni

**Affiliations:** 1 Serum Institute of India Pvt Ltd, Pune, India; 2 Syngene International, Bangalore, India; 3 VisMederi Srl, Siena, Italy

**Keywords:** Live attenuated vaccines, Viral infection

## Abstract

Yellow fever, a mosquito-borne flavivirus infection, is an important public health problem in Africa and Latin America. A Yellow Fever vaccine (YFV) was developed and tested in a study in India. This was a Phase I, open-label, randomized, controlled study where healthy adults received SII YFV intramuscularly (SII YFV IM), SII YFV subcutaneously (SII YFV SC) or STAMARIL^®^ (Sanofi-Pasteur) in 1:1:1 ratio. They were followed for solicited reactions for 10 days and unsolicited events for 28 days and serious adverse events for 3 months. YF-neutralizing antibodies were measured at baseline and on Days 10, 14, 28. A total of 60 adults were enrolled in the study. The proportion of participants with solicited reactions was 10%, 40%, and 25% in SII YFV SC, SII YFV IM, and STAMARIL^®^ arms, respectively. No causally related unsolicited events or any serious adverse event was reported. After vaccination, the seroconversion was 94.44%, 100%, and 100%, in the three arms respectively. The post-vaccination geometric mean titers were similar in the study arms. The new YFV was found safe and immunogenic by IM as well as SC routes. The vaccine can be tested in further phases of clinical studies.

## Introduction

Forty-seven countries in Africa (34) and Central and South America (13) are endemic for yellow fever (YF), and the recent outbreaks are concerning^1^. Annually, YF contributes to 200,000 cases and 30,000 deaths, with 90% contribution from Africa^2^. Thus, countries at highest risk need large-scale, preventive vaccination strategies to establish and maintain high levels of immunity among their populations. YF vaccine (YFV) has been included in routine immunization schedule in 36 of 40 countries at risk of YF in Africa and Americas with an estimated coverage of 47%^3^.

Because of changing epidemiology of YF, a resurgence of mosquitoes, and the risk of international spread^4,5^, not only is there an increased risk of urban outbreaks of YF in the endemic countries but also pose an emerging global threat that requires new strategic thinking. As a result, global demand for YFVs has increased from approximately 20 million doses in 2001 to 90 million doses on average from 2012 onwards. According to the Eliminate Yellow fever Epidemics (EYE) strategy, from 2017 to 2026, the requirement is 1.38 billion doses of YFV for elimination. This growth is mainly due to the demand generated by the resurgence of YF epidemics in Africa and the support provided by Gavi to endemic countries to access the vaccine^6^.

Currently, there are four World Health Organization (WHO) prequalified YF vaccine manufacturers: Sanofi-Pasteur (France), Institut Pasteur de Dakar (Senegal), Bio-Manguinhos (Brazil), and Chumakov Institute (Russian Federation) which produce ~80 million doses annually^6^. This is clearly not enough to meet the increasing demand which has been one of the major obstacles to implementing mass vaccination campaigns, especially in countries with large targeted populations. Vaccine supplies for preventive campaigns have been limited to 15 million people per year, which has led some countries to phase their campaigns over 2 or 3 years, impacting the risk reduction strategy^6^.

Vaccine supply has been continually challenged, mainly by: (i) a sharp increase in demand after the YF investment case; (ii) regulatory and prequalification suspensions; and (iii) production problems, leading to a situation in which supply has been below demand. Vaccine demand is anticipated to continue to outstrip vaccine supply for the immediate future^6^. As a result, the implementation of some preventive mass campaign activities will need to be delayed, spread out, or carried over to subsequent years.

This is evident from WHO and UNICEF 2020 estimates wherein, routine YF vaccination coverage was 44% in the African region, much lower than the 80% threshold required to confer herd immunity against YF. These low YF vaccination coverages indicate the presence of an underlying susceptible population at risk of YF and risk of continued transmission^7^.

Alternative approaches such as subcutaneous administration of fractional dose of YFV have been studied^8^ and successfully implemented in Democratic Republic of Congo^9^ and Brazil^10^ to overcome vaccine shortage. However, usage of fractional doses of YFV is not a long term strategy or to replace the ongoing routine immunization practices, but is to be considered as an emergency response to an outbreak in situations of global shortage of YFV^11^.

Several novel YFVs are in preclinical or early clinical stages of development^12^. However, the challenges of establishing safety and immunogenicity are to be addressed. Live-attenuated YFVs have been used for more than 7 decades with established safety and immunogenicity.

To address this issue of global shortage, a YF vaccine (SII YFV) containing 17D-213 vaccine strain has been developed in India. The strain is present in an already licensed and prequalified vaccine. The safety of SII YFV was demonstrated in six toxicology studies in mice, rats, and monkeys. Following this, a Phase I study was conducted to evaluate the safety and immunogenicity in healthy adult volunteers.

## Results

### Participants

**Fig. 1:**
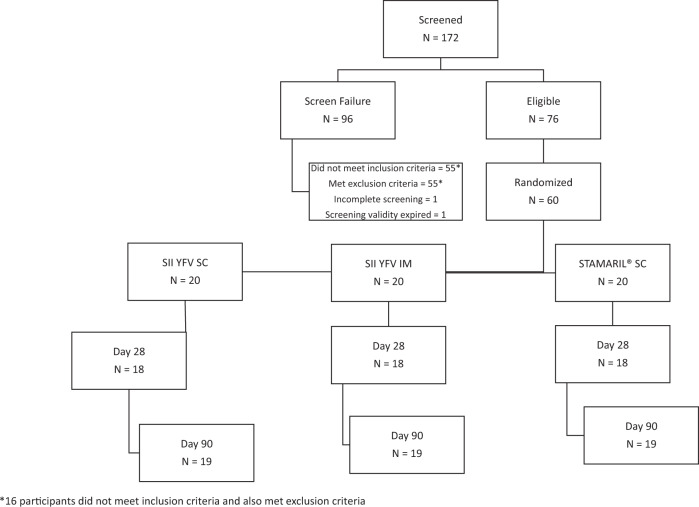
Flow diagram of trial participants. Screening, randomization and follow-up of enrolled participants. Twenty participants were randomized to each group. All participants received a single dose of the study vaccine as per allocated vaccine group. Nineteen participants in each group completed the Day 90 follow-up.

A total of 172 participants were screened, of which 96 were screen failures. Among the screen failures, 55 participants did not meet all inclusion criteria, 55 participants met at least one exclusion criterion, 1 participant went underwent incomplete screening and one participant crossed the screening validity period of 14 days. The inclusion criteria not met were the presence of normal health as determined by medical history, clinical examination, and laboratory assessment (*n* = 53), age criteria of 18–45 years (*n* = 2), and negative urine pregnancy test (*n* = 1). The exclusion criteria met were positive ELISA for YF antibodies (*n* = 41), abnormal ECG or Chest X-ray (*n* = 11), reactive serology to HIV, HbsAg, and hepatitis C (*n* = 2), and investigator’s opinion concerning safety of participant (*n* = 2). Some participants did not meet more than one eligibility criteria. Owing to high screen failure, participants were screened in excess. Out of 76 eligible participants, only 60 were randomized and vaccinated as per study requirements. Fifty-seven participants completed the study. Three participants, one in each group, were lost to follow-up. (Fig. 1)

Baseline characteristics were similar across the groups (Table 1). Most of the participants (86.6%) were males. The mean age at baseline was 30.05, 32.95, and 32.25 years in SII YFV (SC), SII YFV (IM), and STAMARIL^®^ (SC) groups, respectively.

**Table 1:** Baseline Characteristics of Participants.

Parameter	SII YF (SC) *N* = 20	SII YF (IM) *N* = 20	STAMARIL(SC) *N* = 20
Female *n* (%)	2 (10.00)	2 (10.00)	4 (20.00)
Male *n* (%)	18 (90.00)	18 (90.00)	16 (80.00)
Age (In Years)	30.05 ± 7.00	32.95 ± 6.16	32.25 ± 6.75
Weight (Kg)	60.25 ± 5.25	62.00 ± 8.18	60.44 ± 7.03
Height (cm)	163.02 ± 5.62	167.05 ± 7.79	163.82 ± 9.37
BMI (kg/m^2^)	22.68 ± 1.73	22.16 ± 1.82	22.52 ± 1.74
Baseline YF Seropositivity (%)*	1 (5.00)	4 (20.00)	5 (25.00)

Adult participants of either sex, assessed for normal health based on medical history, physical examination and laboratory investigations were enrolled.

*Defined as PRNT ≥ 10 at baseline.

### Safety Results

No immediate AEs were reported in any participant. Two solicited local reactions of pain occurred in 2 (10%) participants, both in the SII YFV (IM) group. Thirty-four solicited systemic reactions occurred in 15 (25%) participants, 6 reactions in 2 (10%) participants in SII YFV (SC), 21 reactions in 8 (40%) participants in SII YFV (IM), and 7 reactions in 5 (25%) participants in STAMARIL^®^ (SC) group. The most common solicited systemic reactions were myalgia, rash, asthenia, arthralgia, and headache. The solicited reactions were mild to moderate in severity and resolved without sequelae (Table 2).

**Table 2:** Solicited reactions and unsolicited adverse events reported by participants.

Description	SII YFV (SC) (*N* = 20) *n* (%), *E*	SII YFV (IM) (*N* = 20) *n* (%), *E*	STAMARIL (SC) (*N* = 20) *n* (%), *E*
**Solicited local reactions**	-	**2 (10), 2**	-
Pain	-	2 (10), 2	-
**Solicited systemic reactions**	**2 (10), 6**	**8 (40), 21**	**5 (25), 7**
Myalgia	2 (10), 2	5 (25), 5	-
Asthenia	2 (10), 2	3 (15), 3	1 (5), 1
Arthralgia	1 (5), 1	4 (20), 4	1 (5), 1
Headache	1 (5), 1	6 (30), 6	3 (15), 3
Nausea	-	-	1 (5), 1
Vomiting	-	1 (5), 1	-
Rash	-	2 (10), 2	1 (5), 1
**Unsolicited adverse events**	-	**4 (20), 6**	**2 (10), 2**
Gastroenteritis	-	1 (5), 1	-
Upper respiratory tract infection	-	1 (5), 1	-
Blood glucose increased	-	1 (5), 1	-
Hepatic enzyme increased	-	1 (5), 1	-
Rash	-	2 (10), 2	2 (10), 2

Solicited reactions and unsolicited events were more in the SII YFV (IM) group compared to the SII YFV (SC) and STAMARIL® (SC) groups.

*n* (%) number and percentage of participants with events, *E* number of events.

Eight unsolicited AEs occurred in 6 (10%) participants, six events in SII YFV (IM), and two events in STAMARIL^®^ (SC) group. These included rash, gastroenteritis, upper respiratory tract infection, increased blood glucose, and increased hepatic enzyme. All the unsolicited AEs were unrelated to the study vaccines. The events were either mild or moderate in severity and recovered without sequelae.

No serious adverse events were reported. Except for two cases (increased blood glucose, and increased hepatic enzyme as mentioned above), the hematology, biochemistry, and urine analysis results were normal in all groups.

### Immunogenicity results

There was a significant increase in geometric mean titer (GMTs) post vaccination as compared to the baseline, with peak GMTs on Day 28. The GMTs in the three groups were more than 5000 in the three groups on Day 28. Seroconversion was reported in all participants on Day 28, except 1 participant in the SII YFV (SC) group. All participants reported seroprotection on Day 28. The post vaccination immune responses in baseline YF seronegative population were similar to that of the overall population. (Table 3).

**Table 3:** Immunogenicity results of participants on Days 1, 10, 14 and 28 of vaccination.

Groups	SII YFV (SC)	SII YFV (IM)	STAMARIL(SC)
Overall population (Per protocol)			
*N*	18	18	18
GMT (95% CI)			
Day 1	5.49 (4.51, 6.69)	6.62 (4.64, 9.46)	8.29 (5.18, 13.29)
Day 10*	13.99 (7.36, 26.59)	15.59 (8.60, 28.28)	36.50 (18.81, 70.81)
Day 14*	350.99 (111.71, 1102.81)	336.29 (107.39, 1053.13)	2401.24 (783.75, 7356.93)
Day 28	7022.68 (2925.21, 16859.64)	14468.62 (10259.31, 20404.98)	5647.48 (2333.90, 13665.51)
Seroconversion *N* (%) (95% CI)			
Day 10*	7 (38.89) (17.30, 64.25)	6 (33.33) (13.34, 59.01)	9 (50) (26.02, 73.98)
Day 14*	15 (83.33) (58.58, 96.42)	17 (94.44) (72.71, 99.86)	18 (100) (81.47, 100)
Day 28	17 (94.44) (72.71, 99.86)	18 (100) (81.47, 100)	18 (100) (81.47, 100)
Seroprotection *N* (%) (95% CI)			
Day 1	1 (5.56) (0.14, 27.29)	3 (16.67) (3.58, 41.42)	5 (27.78) (9.69, 53.48)
Day 10*	8 (44.44) (21.53, 69.24)	10 (55.56) (30.76, 78.47)	14 (77.78) (52.36, 93.59)
Day 14*	16 (88.89) (65.29, 98.62)	18 (100) (81.47, 100)	18 (100) (81.47, 100)
Day 28	18 (100) (81.47, 100)	18 (100) (81.47, 100)	18 (100) (81.47, 100)
Baseline YF seronegative population (modified per protocol)			
*N*	17	15	13
GMT (95% CI)			
Day 1	5 NC	5 NC	5 NC
Day 10*	12.08 (6.61, 22.08)	14.20 (6.96, 28.96)	31.02 (12.91, 74.53)
Day 14*	378.53 (111.83, 1281.30)	342.48 (102.09, 1148.94)	2147.49 (515.85, 8939.97)
Day 28	9025.75 (4291.32, 18983.50)	13800.65 (9116.06, 20892.56)	5484.73 (1918.15, 15682.98)
Seroconversion *N* (%) (95% CI)			
Day 10*	6 (35.29) (14.21, 61.67)	6 (40.00) (16.34, 67.71)	9 (69.23) (38.57, 90.91)
Day 14*	14 (82.35) (56.57, 96.20)	15 (100) (78.20, 100)	13 (100) (75.29, 100)
Day 28	17 (100) (80.49, 100)	15 (100) (78.20, 100)	13 (100) (75.29, 100)
Seroprotection *N* (%) (95% CI)			
Day 1	0 (0) (0, 19.51)	0 (0) (0, 21.80)	0 (0) (0, 24.71)
Day 10*	7 (41.18) (18.44, 67.08)	7 (46.67) (21.27, 73.41)	9 (69.23) (38.57, 90.91)
Day 14*	15 (88.24) (63.56, 100.00)	15 (100) (78.20, 100)	13 (100) (75.29, 100)
Day 28	17 (100) (80.49, 100)	15 (100) (78.20, 100)	13 (100) (75.29, 100)

A gradual increase in GMT were observed in all groups, with highest titres at Day 28. All participants had seroprotective titres at Day 28.

*Two participants missed visits on Days 10 and 14 in SII YFV(SC) and 1 participant missed visit on Day 10 in SII YF (IM) group. Percentages are calculated with total *N* at Day 1 as denominator. *N* number of participants, *NC* not calculated, *CI* confidence intervals.

The GMT and GMT ratios at Day 28 in the flavivirus-naive population were higher compared to the flavivirus-exposed population. The seroconversion and seroprotection rates in both sub-groups were comparable. (Table 4).

**Table 4:** Day 28 immunogenicity results in sub-groups based on flavivirus exposure status (Per Protocol population).

Groups	SII YFV (SC)		SII YFV (IM)		STAMARIL (SC)	
	Flavivirus exposed (*N* = 12)	Flavivirus naive (*N* = 5)	Flavivirus exposed (*N* = 11)	Flavivirus naive (*N* = 7)	Flavivirus exposed (*N* = 14)	Flavivirus naive (*N* = 4)
GMT (95% CI)	6632.89 (1858.28, 23675.29)	12487.57 (5070.64, 30753.35)	13057.07 (7637.51, 22322.33)	17001.37 (10781.05, 26810.62)	4437.43 (1442.98, 13645.90)	13133.67 (5757.07, 29962.01)
GMT ratio to baseline (95% CI)	1153.12 (258.71, 5139.67)	2497.51 (1014.13, 6150.67)	1755.45 (849.51, 3627.54)	3079.71 (1907.66, 4971.86)	504.11 (150.75, 1685.77)	1950.96 (853.26, 4460.82)
Seroconversion *N* (%) (95% CI)	11 (91.67) (61.52, 99.79)	5 (100) (47.82, 100)	11 (100) (71.51, 100)	7 (100) (59.04, 100)	14 (100) (76.84, 100)	4 (100) (39.76, 100)
Seroprotection *N* (%) (95% CI)	12 (100) (73.54, 100)	5 (100) (47.82, 100)	11 (100) (71.51, 100)	7 (100) (59.04, 100)	14 (100) (76.84, 100)	4 (100) (39.76, 100)

Flavivirus naive participants had higher GMT and GMT ratios (to baseline) compared to flavivirus exposed participants. All participants were seroprotected at Day 28 irrespective of the flavivirus exposure status.

*N* number of participants, *CI* confidence intervals.

## Discussion

This was a Phase 1, randomized controlled study on the SII YFV (SC and IM) compared to STAMARIL^®^ in 60 adults. The vaccine was found highly immunogenic with both routes. The antibody titers were comparable between the vaccines. The vaccine was also safe and well tolerated. The AEs were mostly mild and transient.

Phase I was conducted in India, as it is non-endemic for YF, and the vaccine response would not be affected by the pre-existing YF antibodies. This was further ensured by screening the participants for YF IgG antibodies. However, there were several screen failures due to positive YF IgG antibodies. This was surprising as India is not endemic for YF and screen failures had not received YF vaccines nor had they visited endemic countries. India is endemic for other flavivirus infections (dengue and Japanese encephalitis) and there could be cross-reactivity with the serological tests for YF.

SII YFV was evaluated by both SC and IM routes. The Russian vaccine is given by both SC and IM routes^13^. While STAMARIL^®^ is recommended by SC route^14^. Though most of the YFV can be given by both routes, there is a lack of data that compares both routes head-to-head. To our knowledge, such head-to-head comparison studies of SC and IM routes of YF vaccines are not available in recent decades, since 1943^15^. The present study is the first such evidence and demonstrates that the immune responses by both routes are comparable.

The study showed that the SII YFV was safe by both SC and IM routes. The incidence of solicited reactions seems comparatively higher in the SII YFV (IM) group when compared to SII YFV SC and STAMARIL^®^. However, the sample size is small and not powered for such comparisons.

The immune responses showed that the SII YFV was highly immunogenic by both SC and IM routes and the GMTs in both groups were comparable to STAMARIL^®^. We checked titers on days 10, 14, and 28 and found that the GMTs increased gradually with the highest titers on Day 28. This is known with other YFVs^16,17^. Seroconversion and seroprotection were seen in almost all participants in SII YFV groups similar to STAMARIL^®^.

We conducted a modified PP analysis that comprised of baseline YF-neutralizing antibody seronegative population. We did not observe differences in the immune responses in the baseline seronegative vs overall populations, as the immune responses to the YF vaccine were more than 1000 fold post-vaccination in both populations.

The flavivirus-naive status of participants was based on pre-existing antibodies to Dengue and JE which are endemic in India. The previous flavivirus exposure may interfere with the YFV immune response^18^. In the present study, the titers were numerically higher (though not significant) in the naive than the exposed group, except in the SII YFV IM group though the study is not powered for such comparison. However, this interference may not be of clinical significance as the absolute values of GMTs are very high in all three groups (>4400). This is important as the YFV will be used in countries that have several circulating flavivirus infections.

One limitation of our study is that it is an open-label study. There were two reasons for this: SII YFV and STAMARIL^®^ have distinct appearances and secondly two different routes were being evaluated. Both factors would have made it difficult to blind the SII YFV (IM) group, as the control vaccine was administered by SC route only. In any case, the laboratory personnel was blind to study assignments, and hence, we believe the open-label design has not led to any bias.

To conclude, a single dose of SII YFV was found safe, well tolerated, and immunogenic by two routes in adults. Based on these positive results, two Phase 3 clinical studies are planned in sub-Saharan countries in infants, children, and adults. This vaccine may eventually help address the global shortage of YFVs.

## Methods

### Study design

This was a Phase I, open-label, single-center, randomized, active-controlled, parallel group study. Participants were randomized to the three study groups in a ratio of 1:1:1 and administered a single dose of either SII YFV (SC route) or SII YFV (IM route) or STAMARIL^®^ (SC route), respectively.

### Study participants

60 healthy Indian adults (18–45 years) were enrolled at Human Pharmacology Unit - Syngene International Limited, Bangalore between October 2020 and February 2021. Individuals with fever, or any acute infection were temporarily excluded. Other key exclusion criteria were: known hypersensitivity to any of the vaccine components (including gelatin, eggs, egg products, or chicken protein) or to a vaccine containing the same substances; previous vaccination or infection with YF, tick-borne encephalitis (TBE), Japanese encephalitis virus (JE) or dengue fever, West Nile Virus (WNV); travel to a YF endemic area; positive ELISA for YF virus antibodies; pregnant or lactating women; immunocompromised status.

### Study products

*SII YFV* contains the live-attenuated 17D-213 vaccine strain, a derivative of 17D-204 strain, in a lyophilized formulation and based on specific pathogen-free embryonated hen’s egg. A single dose vial presentation (Batch no. 317003, Expiry Mar. 2021, virus concentration 4.242 log10 IU per human dose) was used. After reconstitution with sterile water for injection, one dose (0.5 mL) contains yellow fever virus not <1000 IU.

*STAMARIL*^®^ (Batch no R3N044V, Expiry Oct. 2021) was used as a WHO-prequalified control vaccine. It contains, a live, attenuated, freeze-dried (lyophilized) vaccine (17D-204 strain not less than 1000 IU/dose) in a single dose vial and is reconstituted with provided solvent (0.9% sodium chloride solution) in a prefilled syringe.

These vaccines were administered as a single dose by route as per the randomization.

### Safety assessments

Participants were observed post vaccination for at least 1 hour for any immediate AEs and were followed on Days 10, 14, 28, and 90 for safety assessments. At every visit, inquiry was made for AEs and they were physically examined. Safety laboratory tests (hematology, biochemistry, and urinalysis), were assessed at screening and on Day 28 post vaccination.

Active surveillance for vaccine reactogenicity over the 10-day post vaccination period was conducted using a diary card. These included: injection site redness, pain, induration; fever, myalgia, asthenia, arthralgia, headache, nausea, vomiting, and rash. Surveillance for unsolicited AEs was carried out till 28 days post vaccination using a diary card.

Serious adverse events were looked for 90 days post vaccination.

### Immunogenicity assessments

Immune response against Yellow Fever virus was measured by a validated Plaque Reduction Neutralization Test (PRNT50) at baseline, Days 10, 14, and 28. The tests were performed at VisMederi Srl, Siena, Italy.

Procedure - Vero E6 cells (ATCC CRL-1586) were maintained in DMEM High Glucose supplemented with 10% heat-inactivated FBS + 1% Penicillin- Streptomycin (100 U/ml final concentration), 1% L-glutamine (2 mM final concentration). The assay was performed in duplicate using 6-well plates (with 6.7 × 10^5^ cell/ml, seeded in 1.5 ml) in a biosafety level 3 facility. Serial dilutions of each serum sample were incubated with the virus solution for 1 h at 37 °C. The final virus concentration used, was approximately 50 PFU/0.2 ml. The virus-serum mixtures were inoculated onto pre-formed Vero E6 cell monolayers and incubated for 1 h at 37 °C in 5% CO2. After the incubation the inoculum was removed and the cell monolayer was then overlaid with the Overlay medium composed by a Serum diluent medium (EMEM supplemented with 10% heat-inactivated FBS, 2% Penicilin-Streptomycin, 1% L-glutamine, 2% HEPES, 2% NEAA) and an equal volume of 2% agarose solution. After 5 days of incubation at 37 °C in 5% CO2, the plates were stained with a second overlay, containing Overlay medium and neutral red at 0.05% final concentration. Plaques were manually counted 18-20 hours later from the staining with the second overlay and PRNT50 was determined using a vectorial form. Positive Control (procured by NIBSC code:YF) was used in validation experiments, and added in each experiment session. The viral strain used is the Yellow Fever 17D.

Seroconversion was defined as four-fold or more increase in neutralizing antibody levels with respect to baseline. Seroprotection was defined as a neutralizing antibody titer ≥ 1:10.

Further, to assess prior Flavivirus exposure, at baseline, participants were assessed for Dengue and Japanese encephalitis IgG antibodies, by ELISA.

### Randomization

Participants were randomized in the three groups in equal allocation by applying permutated block randomization procedure. The randomization schedule was generated by using PROC PLAN procedure of SAS^®^ 9.4 (SAS institute Inc, USA).

### Statistical analysis

The statistical analyses were performed using SAS^®^ version 9.4.

*Per Protocol (PP) Population* included all randomized participants who received the study vaccine, had given blood samples at baseline and on 28 (+7) days post vaccination and without any major protocol deviation.

*Modified Per Protocol (mPP) Population* included all randomized participants who received the study vaccine, had given blood samples at baseline and on 28 (+7) days post vaccination, were seronegative (PRNT50 < 1:10) to Yellow fever at baseline and without any major protocol deviation.

AEs were reported as a percentage of participants with events and, E was the number of events in participants.

The GMT of PRNT50 was assessed for each group along with two-sided exact 95% confidence intervals. Seroconversion and seroprotection for each group were calculated along with two-sided exact 95% confidence intervals based on the Clopper–Pearson method. The above variables were also calculated for sub-groups based on prior flavivirus exposure.

### Reporting summary

Further information on research design is available in the Nature Research Reporting Summary linked to this article.

## Supplementary information

REPORTING SUMMARY

## Data Availability

Data supporting the study observations are available from the corresponding author upon request.

## Regulatory compliance

The study was approved by the Drugs Controller General of India. The study is registered on the clinical trial registry of India (CTRI/2020/09/027594).
