# Supplementary material for: A phase I clinical study to assess safety and immunogenicity of yellow fever vaccine
Source: NPJ Vaccines. 2022 Dec 19;7:170. doi: 10.1038/s41541-022-00595-6 (PMC9763341; doi:10.1038/s41541-022-00595-6)
Supplement: Supplementary file 1 — REPORTING SUMMARY [file 41541_2022_595_MOESM1_ESM.pdf]

nature portfolio

Corresponding author(s): Dr Prasad Kulkarni

Last updated by author(s): Nov 9, 2022

Reporting Summary

Nature Portfolio wishes to improve the reproducibility of the work that we publish. This form provides structure for consistency and transparency in reporting. For further information on Nature Portfolio policies, see our Editorial Policies and the Editorial Policy Checklist.

Statistics

For all statistical analyses, confirm that the following items are present in the figure legend, table legend, main text, or Methods section.

n/a	Confirmed	
☐	☒	The exact sample size (*n*) for each experimental group/condition, given as a discrete number and unit of measurement
☐	☒	A statement on whether measurements were taken from distinct samples or whether the same sample was measured repeatedly
☐	☒	The statistical test(s) used AND whether they are one- or two-sided<br>*Only common tests should be described solely by name; describe more complex techniques in the Methods section.*
☒	☐	A description of all covariates tested
☒	☐	A description of any assumptions or corrections, such as tests of normality and adjustment for multiple comparisons
☐	☒	A full description of the statistical parameters including central tendency (e.g. means) or other basic estimates (e.g. regression coefficient) AND variation (e.g. standard deviation) or associated estimates of uncertainty (e.g. confidence intervals)
☒	☐	For null hypothesis testing, the test statistic (e.g. *F*, *t*, *r*) with confidence intervals, effect sizes, degrees of freedom and *P* value noted<br>*Give P values as exact values whenever suitable.*
☒	☐	For Bayesian analysis, information on the choice of priors and Markov chain Monte Carlo settings
☒	☐	For hierarchical and complex designs, identification of the appropriate level for tests and full reporting of outcomes
☒	☐	Estimates of effect sizes (e.g. Cohen's *d*, Pearson's *r*), indicating how they were calculated

Our web collection on statistics for biologists contains articles on many of the points above.

Software and code

Policy information about availability of computer code

Data collection	Data collection was done using electronic CRF on Techsol (Oracle)
Data analysis	Statistical Analysis was performed using SAS version 9.4

For manuscripts utilizing custom algorithms or software that are central to the research but not yet described in published literature, software must be made available to editors and reviewers. We strongly encourage code deposition in a community repository (e.g. GitHub). See the Nature Portfolio guidelines for submitting code & software for further information.

Data

Policy information about availability of data

All manuscripts must include a data availability statement. This statement should provide the following information, where applicable:

- Accession codes, unique identifiers, or web links for publicly available datasets
- A description of any restrictions on data availability
- For clinical datasets or third party data, please ensure that the statement adheres to our policy

Data supporting the study observations are available from the corresponding author upon request.

March 2021

Human research participants

Policy information about studies involving human research participants and Sex and Gender in Research.

Reporting on sex and gender	This was a Phase I study of SII Yellow Fever vaccine and included representation from both genders at the time of screening. On the completion of study, majority (86.6%) of participants were males.
Population characteristics	60 healthy Indian adults (18-45 years) of either sex were enrolled
Recruitment	Participants were recruited from the participant database at Human Pharmacology Unit - Syngene International Limited, Bangalore, India
Ethics oversight	The study protocol and documents were approved by the People Tree Hospital Ethics Committee.

Note that full information on the approval of the study protocol must also be provided in the manuscript.

Field-specific reporting

Please select the one below that is the best fit for your research. If you are not sure, read the appropriate sections before making your selection.

☒ Life sciences ☐ Behavioural & social sciences ☐ Ecological, evolutionary & environmental sciences

For a reference copy of the document with all sections, see nature.com/documents/nr-reporting-summary-flat.pdf

Life sciences study design

All studies must disclose on these points even when the disclosure is negative.

Sample size	As this was a Phase I study, no formal sample size calculation was performed.
Data exclusions	Key exclusion criteria were: fever, or any acute infection, known hypersensitivity to any of the vaccine components (including gelatin, eggs, egg products, or chicken protein) or to a vaccine containing the same substances; previous vaccination or infection with YF, tick-borne encephalitis (TBE), Japanese encephalitis virus (JE) or dengue fever, West Nile Virus (WNV); travel to a YF endemic area; positive ELISA for YF virus antibodies; pregnant or lactating women; immunocompromised status. No data was excluded from analysis
Replication	PRNT testing of each sera sample was tested in duplicate
Randomization	Participants were randomized in the three groups in equal allocation by applying permutated block randomization procedure. The randomization schedule was generated by using PROC PLAN procedure of SAS® 9.4 (SAS institute Inc, USA).
Blinding	This was an open label study

Reporting for specific materials, systems and methods

We require information from authors about some types of materials, experimental systems and methods used in many studies. Here, indicate whether each material, system or method listed is relevant to your study. If you are not sure if a list item applies to your research, read the appropriate section before selecting a response.

Materials & experimental systems

n/a	Involved in the study	
☒	☐	Antibodies
☒	☐	Eukaryotic cell lines
☒	☐	Palaeontology and archaeology
☒	☐	Animals and other organisms
☐	☒	Clinical data
☒	☐	Dual use research of concern

Methods

n/a	Involved in the study	
☒	☐	ChIP-seq
☒	☐	Flow cytometry
☒	☐	MRI-based neuroimaging

Clinical data

Policy information about clinical studies

All manuscripts should comply with the ICMJE guidelines for publication of clinical research and a completed CONSORT checklist must be included with all submissions.

Clinical trial registration	CTRI/2020/09/027594
Study protocol	Study protocol is available on ctri.nic.in and can be made available to interested parties on request
Data collection	Participants were enrolled at Human Pharmacology Unit - Syngene International Limited, Bangalore between October 2020 and February 2021
Outcomes	Immune response against Yellow Fever virus was measured by a validated Plaque Reduction Neutralization Test (PRNT50) at baseline, Days 10, 14, and 28. Participants were observed post-vaccination for at least one hour for any immediate AEs and were followed on Days 10, 14, 28 and 90 for safety assessments. Active surveillance for vaccine reactogenicity over the 10-day post-vaccination period for solicited events: Injection site redness, pain and induration, fever, myalgia, asthenia, arthralgia, headache, nausea, vomiting and rash. Surveillance for unsolicited AEs was carried out till 28 days post-vaccination. Serious Adverse Event (SAE) were looked for 90 days post-vaccination.
